# Exploring Access as a Process of Adaptation in a Self-Monitoring Perinatal eHealth System: Mixed Methods Study From a Sociomaterial Perspective

**DOI:** 10.2196/44385

**Published:** 2023-05-15

**Authors:** Jennifer Auxier, Kaisu T Savolainen, Miriam Bender, Amir M Rahmani, Fatemeh Sarhaddi, Iman Azimi, Anna M Axelin

**Affiliations:** 1 Department of Nursing Science Faculty of Medicine University of Turku Turku Finland; 2 Sue & Bill Gross School of Nursing University of California, Irvine Irvine, CA United States; 3 Donald Bren School of Information and Computer Sciences University of California, Irvine Irvine, CA United States; 4 Department of Future Technologies University of Turku Turku Finland

**Keywords:** patient engagement, eHealth, pregnancy, motivation, pregnant, maternal, cocreation, participatory, codesign, use pattern, usage, self-monitor, sociomaterial

## Abstract

**Background:**

The development and quality assurance of perinatal eHealth self-monitoring systems is an upcoming area of inquiry in health science. Building patient engagement into eHealth development as a core component has potential to guide process evaluation. Access, 1 attribute of patient engagement, is the focus of study here. Access to eHealth self-monitoring programs has the potential to influence pregnancy health and wellness outcomes. Little is known about how pregnant users’ ability to obtain resources is influenced by their own adaptive activities and the mediating activities of eHealth systems during the process of real-world testing of these systems.

**Objective:**

Here, we examine the patient engagement process of access occurring during the adaptation of eHealth self-monitoring use from a sociomaterial perspective.

**Methods:**

In this mixed methods convergent evaluation design, we interviewed women about perceptions of the adaptation process of using an eHealth self-monitoring system. Deductive analysis was conducted guided by the definition of access as an attribute of patient engagement. After initial qualitative and quantitative data collection and analysis, participants were spilt based on their level of use of the eHealth system (physical wear time of self-monitoring device). Content analysis was then conducted according to user group, using a conceptual matrix developed from ontological perspectives of sociomateriality.

**Results:**

Pregnant users’ adaptive activities and the mediation activities of the eHealth system represent a cocreation process that resulted in user group–specific characteristics of accessing and using the system. The high- and low-use groups experienced different personal adaptation and eHealth mediation during this process of cocreation. Differences were noted between high- and low-use groups, with the high-use group giving attention to developing skills in recording and interpreting data and the low-use group discussing the manual adding of activities to the system and how the system worked best for them when they used it in their mother tongue.

**Conclusions:**

A cocreation process between pregnant users and the eHealth system was identified, illustrating access as a useful core component of perinatal eHealth self-monitoring systems. Researchers and clinicians can observe reasons for why pregnant users access eHealth systems in unique ways based on their personal preferences, habits, and values. Mediation activities of the eHealth system and the different user adaptive activities represent a cocreation process between the users and the eHealth system that is necessary for the personalization of perinatal eHealth systems.

## Introduction

### Background

Pregnancy is a time when persons and women think about their own health as it relates to their unborn child and sometimes use this period to form new health-related habits [1]. eHealth resources are becoming a common source of support for health care users. The use of eHealth resources has been associated with individual motivations for improving health and lifestyle [2], improving health literacy [2], and having enough time and choice to use the resources tailored to their personal lifestyle and circumstances [3]. Although we understand the circumstances in which pregnant persons and women are likely to use resources when access is given to them, knowledge about how their choice of use leads them to being able to obtain the needed resources and support required for the maintenance of health and healthy lifestyles during pregnancy has been less studied. Exploring user behaviors that impact habituation or meaningful use of eHealth self-monitoring systems could provide insights into why certain individuals use devices more than others and whether the use is connected to appropriate receival of health resources.

The provision of accessible eHealth is a global concern. Access can be facilitated by designing systems that are easy to use, are convenient, and negate travel to clinic or hospital settings [4]. Barriers to supporting accessible eHealth systems are improperly matched technology, low eHealth literacy, and a lack of financial or structural resources supporting eHealth systems [4]. Access to eHealth during pregnancy has been associated with increased engagement in self-management tasks and antenatal clinic visits, and satisfaction with care [5-7]. What is less understood are the processes under which pregnant persons and women become accustomed to using eHealth resources and discover their patterns of use for meaningful engagement.

Higgins et al [8] define patient engagement as a behavior and a process within health care. Patient engagement is a multifaceted concept with 4 attributes: personalization, therapeutic alliance, commitment, and access [8]. Access is defined as “…the ability of [individuals] to obtain information, guidance, and tools to secure consistent, high quality, [and] appropriate care” [8]. Access has been mainly studied from the perspective of adherence and the frequency of use rather than from a process perspective. Exploring the interactions and impact of the interrelated processes that occur once access to eHealth is given could inform nuances of appropriate care/support receival. Perinatal eHealth feasibility studies conducted in United States and the Netherlands have illustrated that trusting interactions between health providers and clients are a component of appropriate antenatal care, as well as the presence of a shared understanding of current health states of pregnant users and their care providers [9,10]. eHealth self-monitoring is being applied in the United Kingdom to support trusting relationships and foster honest reporting of health states [11]. Less is known about pregnant persons’ personal motivations for engaging in self-monitoring when eHealth is made accessible to them. With the availability of an eHealth self-monitoring system, it is expected that users choose eHealth features that they grow to identify with [12]. Little is known about perinatal user-eHealth interactions and technological mediations that occur during real-world testing of eHealth self-monitoring during pregnancy.

With the use of an available smart-ring and wellness app, ŌURA, pregnant users can track their total sleep time and sleep cycles, resting heart rate and variability, daily activity levels, and physical wear time of the device [13]. ŌURA provides an opportunity to view personal health data, health status alerts (eg, not enough total sleep), and recommendations for level of physical activity based on the previous night’s sleep and day’s activity levels. Users can set goals with the support of tailored feedback and health data trends.

### Objective

The objective of this study was to examine the processes occurring during the adapting of eHealth self-monitoring use, with a focus on the technology-pregnant user interactions. To that end, our research question was developed using the sociomaterial perspective and became, What are the characteristics of the process of use after pregnant users receive access to an eHealth self-monitoring system?

## Methods

### Study Design

A mixed methods convergent evaluation design was conducted, whereby data were collected at parallel time points and brought together during the analysis step of the study [14]. Participants collected use data (according to wear time) throughout the pilot testing of the self-monitoring system and participated in semistructured exit interviews. The full data set was examined for elements related to the process of accessing the eHealth self-monitoring system. See Table 1 for stages of data convergence.

**Table 1:** Concepts under study and convergence of data sources.

Method	Concept under study	Convergence of data
Qualitative: semistructured interviews with pregnant users about their perceptions of access	Access as a process attribute of patient engagement: “…the ability of [individuals] to obtain information, guidance, and tools to secure consistent, high quality, appropriate care” [8].	Interview data related to access of eHealth self-monitoring system modalities After group stratification, analysis to identify pregnant users and eHealth system mediating activities according to user group
Quantitative: distribution of nonwear time of pregnant users recorded with the ŌURA wearable ring that pregnant users wore throughout the pilot use of the self-monitoring wellness eHealth system	Wear time measurement and group stratification allowed us to examine differences in the level of use.	User groups identified through the kernel density estimate test [15]

The sociomaterial perspective was used as a theoretical frame of this study. This perspective proposes that an interconnectedness exists between technology, work, and organizations [16]. This perspective supports the nonhierarchical relationship between technology and humans, wherein both humans and technology play a role in the creation of social and societal processes [16]. This nonhierarchical relationship between objects and humans can also be understood through an ontology of mutually dependent ensembles, as described by Orlikowski and Scott [16]. The pervasive presence of a new technology is not only meaningful at specified events or processes within a health program but also provides mediation and emergence of patterns of accessing services during program delivery [16]. Activities of pregnant users will be mediated by new technologies, and the accessibility of these systems may be understood through the eHealth mediation activities and the responsiveness of the pregnant persons during their engagement with the systems. The sociomaterial perspective supports examining pregnant users’ activities after their newly acquired access to an eHealth lifestyle self-monitoring system.

### Setting and Participants

In total, 20 pregnant women in their second trimester were sampled, using convenience sampling, to take part in the pilot use of an eHealth self-monitoring system [17]. Two perinatal clinics in Raisio and Rusko and their public health nurses (PHNs) were willing to participate in the pilot use of the ŌURA smart ring and wellness app. The 2 public clinics represent 2 separate but financially linked health service organizations. Inclusion criteria were pregnant users being 18 years of age or older, in the second trimester of pregnancy, having access to a smartphone (Android or iOS), and understanding Finnish and English languages. Pregnant users were recruited from early antenatal visits during their second trimester by the PHNs. Refer to Auxier et al [18] for details regarding recruitment and informed consent.

### Pilot Testing of the eHealth Self-Monitoring System

The ŌURA ring is a commercial smart ring that collects heart rate and variability, sleep, body temperature, respiratory rate, and physical activity data. Previous studies with nonpregnant persons indicated the reliability of self-monitoring with ŌURA, including heart rate and variability, sleep, and physical activity [19-21]. The ring is small, lightweight, and easy to use for long-term monitoring. It has up to 1-week battery life and sends the collected data via a Bluetooth connection to a mobile app and cloud server (iOS and Android). The ŌURA app comprises a main panel that presents the user’s daily sleep, activity, and a proprietary readiness score. Users can view their daily and weekly trends on the mobile app and are able to download personal data and view monthly and yearly trends from a web app on a desktop or laptop computer. The collected data can be visualized on smartphones and computers. The data were collected and stored using anonymous usernames. Data are computed for activity, sleep, and readiness scores based on previous data. Personalized feedback, recommendations, and goal adjustments to balance the activity and rest are then provided by the app on demand and in real time. Women wore the ŌURA ring and used the wellness app to track and view their wellness data for an average of 9.5 weeks, with women having 2-4 visits with PHNs over the course of the study. The PHNs were trained to provide their normal wellness coaching during visits but could use the personal data that women had available from the wellness app to inform their regular perinatal coaching if they saw an opportunity to do so. The PHNs were given an ŌURA ring and shown how to use the app for themselves and could contact the research nurse assistant at any time for questions or comments regarding the use of the smart ring device, app, or coaching of women during perinatal visits.

### Semistructured Interviews

The interview guide was generated by the first, second, and last authors based on the constructs of patient engagement. The second author met each of the pregnant women at the end of the pilot at a location of their choice where they could participate in 1-on-1 semistructured interviews. Interviews were audio-recorded and transcribed verbatim, and all raw audio and document files were digitized and stored in secure password-protected files. Typed transcripts were cleaned of identifying information prior to analysis.

### Data Analysis

**Figure 1:**
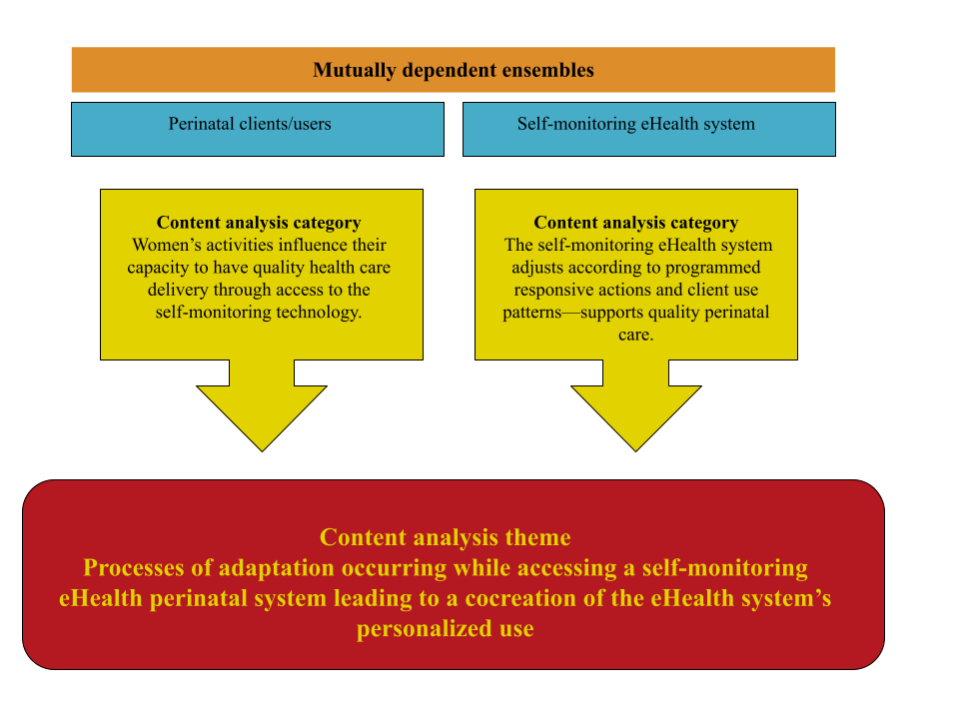
Conceptual matrix using mutually dependent ensembles from the sociomaterial perspective.

The study was conducted guided by a content analysis deductive approach using the definition of access and statistical user group stratification. All qualitative data were collected and analyzed deductively to identify processes related to access prior to group stratification. Participants were stratified according to the distribution of individual means of nonwear time of the smart ring. See further details of stratification reported previously [18]. Convergence of data sources was then conducted by organizing qualitative data according to user group. A final conceptual matrix was constructed using a model of sociomaterial interactions in which the technology in use and the social processes are not seen as separated from each other but part of complex interactive processes (see Figure 1) [16]. This matrix was used once the deductive and inductive development of codes and categories revealed a fit between the main domains and a sociomaterial perspective [16,22,23]. A description of the quantitative concept (wear time) and qualitative concept (access as a process) can be seen in Table 1.

### Ethical Considerations

This study was accepted by the Ethics Committee of the Hospital District of Southwest Finland (approval ID: ETMK Dnro: 1/1801/2020). Each participant provided written informed consent before participation in the study and was aware that they could exit the study at any time.

## Results

### Participants

In total, 20 pregnant women monitored themselves in either a high (n=14, 70%) or a low (n=6, 30%) amount (Table 2) based on the kernel density estimate conducted in our previous study [18]. Women described that the eHealth system’s mediating activities and their own adaptive activities of self-monitoring enacted a process of cocreation for eHealth system use. The new interactions that developed in the real-world use of this eHealth system illustrated emerging scenarios and considerations for the receival of appropriate resources. The main theme was the cocreation of eHealth self-monitoring system usage. The eHealth system usage was attained through (1) adaptive activities of the pregnant user and (2) mediation activities of the system. These activities varied in some cases based on user group (see Table 3).

**Table 2:** Baseline characteristics according to group.

Characteristics			High-use group (n=14)		Low-use group (n=6)
Age (years), mean (SD)			32 (2.42)		29 (3.01)
Average gestation (days), mean (SD)			108.86 (12.59)		107.50 (6.66)
Wearing device at work, n (%)			13 (93)		1 (17)
Daily nonwear time (minutes/day), mean (SD)			7.13 (15.35)		32.49 (23.99)
BMI, mean (SD; range)			24.95 (3.49; 17.43-31.64)		26.48 (6.92; 20.96-39.84)
**Number of children, n (%)**					
	1	6 (43)		2 (33)	
	2	3 (21)		1 (17)	
	0	5 (36)		3 (50)	
**Frequency of other mobile app use in daily life, n (%)**					
	Daily	8 (57)		3 (50)	
	Weekly	5 (36)		2 (33)	
	Monthly	1 (7)		0	
	Rarely	0		1 (17)	
**Baseline health survey scores**					
	EPDS^a^ score, mean (SD; range)	4.57 (2.90; 1-12)		3.50 (3.08; 0-8)	
	Perceived stress, mean (SD; range)	37.14 (5.14; 29-44)		37.67 (7.66; 27-46)	
	PRAQ-R^b^, mean (SD; range)	5.57 (0.85; 4-7)		5.67 (0.52; 5-6)	
	SOC-13^c^, mean (SD; range)	72.64 (5.62; 62-81)		77.00 (4.73; 71-84)	

^a^EPDS: Edinburgh Postnatal Depression Scale.

^b^PRAQ-R: Pregnancy-Related Anxiety Questionnaire –Revised

^c^SOC-13: 13-item Sense of Coherence scale.

**Table 3:** Characteristics of adaptive and mediating activities according to user group.

User group	Both groups	High-use only	Low-use only
Adaptive activities of pregnant users	Use related to self-awareness Practical matters of reviewing tips and feedback Use related to comfort and preference, and knowledge	Practical matters of recording daily data	Added their own activities when the eHealth system did not record certain exercises automatically Preferred to use the system in their mother tongue (Finnish)
Mediating activities of eHealth system	Flexible and responsive tips and information New opportunities for participation	Gentle and guiding nature of eHealth advice	Mentioned that nursing guidance and advice were kind and nonjudgmental

### Cocreation of eHealth System Usage Between Pregnant Users and Technology

#### Pregnant User Adaptive Activities

Participants described a process of adapting after receiving access to the eHealth self-monitoring system. The process incorporated personal choices to use the system, depending on eHealth literacy, comfort with the technology, and interest in eHealth, as a tool for health promotion. Participants expressed that personal self-awareness, perceptions of trusting their own bodies, and participation in goal setting activities emerged as important elements of using the eHealth system. Women in the high-use group found themselves becoming focused on the practical skills of monitoring and interpreting data. Unlike the low-use group, this active focus and concentration on maintaining good self-monitoring techniques made it difficult for them to use the data initially in relevant ways for their own health promotion. Women in the low-use group were instead concerned about whether they could incorporate this device into their daily lives, for example, remembering where they put the ring after washing their hands, and whether they would have to remember to manually add their exercises into the app.

#### Use Related to Self-Awareness

Participants began looking at their daily activity and sleep pattern data using the app. Women refer to bodily self-awareness as critical to adapting to the daily viewing of personal health data. Some women continued to be interested in seeing the daily sleep and activity patterns and told the researcher that they liked becoming aware of their own daily habits. Multiple women in the high-use group stated that they never knew they slept poorly before using the service and that after learning this, they gave themselves permission to rest more. One woman commented that she was really trusting the data from the app:

…Somehow it’s also nice to know that maybe my own feeling isn’t always in line with the data. I somehow trust the app and what it says [about my sleep].

Participant 2

Women’s personal preferences, attitudes, and past life experience contributed to their impressions of self-monitoring. Some participants identified as having good self-awareness prior to practicing self-monitoring and felt that listening to their own bodily rhythms was crucial. One woman in the low-use group commented on her experience with physical activity recommendations:

I think the weirdest part was that I don’t feel I exercise a lot, but still [the app] may announce that you have exercised a lot yesterday, so today it’s good for you to take it easy…there might be many days when it announced that you haven’t recovered yesterday so take it easy…[but]…I haven’t done anything heavy. Other than cycled [to work].

Participant 10

The system was not responsive to the bodily changes unique to pregnancy, and some women gave little weight to the interpretations from the app because of this. Many women in the high-use group discussed that they had confidence in their own body rhythms and health habits and felt that the App was used for reassurance, that things were as well as they felt them to be. Some participants also noted that this system would be well suited to persons with some health challenges but maybe not needed for someone like them.

#### Practical Matters of Recording Daily Data: High-Use Group

Some women in the high-use group became distracted with the practical matters of self-monitoring and interpreting data, and in this way, a focus on simple ways of improving activity or sleep quality was not a priority. These women expressed that they were focused on becoming technically skilled at recording their daily data and understanding the functionality of the system. They wondered what to do if the system was making the wrong recordings or wrong conclusions about daily patterns. For example, 1 woman noticed that the system gave a tip that eating before going to bed could prevent her higher resting heart rate at the start of sleep on an evening that she was fasting. The woman was concerned about how to proceed with the wrong messaging.

#### Practical Matters of Reviewing Tips and Feedback

The app was only available in English at the beginning of the feasibility study; however, some women received access to the Finnish version of the app during their use. Women in the low-use group mentioned that this update supported them to identify more with the feedback. The women who found the use of their mother tongue helpful stated that they could internalize the feedback better and that this added clarity to the messages given by the app. One woman mentioned that she explored the definitions and educational sections of the app in greater detail:

I started to use maybe more [information from the app] when the upgrade of the Finnish version came. When the app became Finnish, I was able to look all the different things because some of the [English words] were so specialized.

Participant 10

The app suggests goals for women to make based on their behavior patterns. Multiple participants expressed that after regularly viewing their personal data on activity, sleep, and recovery, they took on an interested passive observer role. They found the patterns interesting to watch and found no reason to make use of the goal-setting function, as 1 woman explained:

I have been a good sleeper always and even after a night shift…[Since using the smart ring] it has been nice to look at it [sleep patterns].

Participant 21

One woman (high-use group) felt her health behaviors were not changeable in anyway because she was pregnant. She thought it was good to just monitor what naturally happened due environmental or other contextual changes in life:

I’ve thought I would buy some smart watch or so, but then after pregnancy, not now when sleeping poorly and exercising so little. But still, it’s interesting to observe the data because it really got better during the summer.

Participant 6

Some participants described times where they made changes to their habits in daily life because of the tips from the app. Women cited changes made to sedentary time, frequency of restful moments, not eating snacks close to going to sleep, and considering new stress management strategies.

#### Use Related to Comfort, Preference, and Knowledge

When the data recordings were not in agreement with women’s own bodily perceptions or knowledge of events, they often referred less to the app for information. One woman explained her habit of examining the recovery score from the app over time:

At first, I checked that state of readiness, but I’m not sure, because I feel it’s not reflecting my real feeling. Sometimes it said the readiness was at its highest and I felt that no, not today.

Participant 3

Some women in the low-use group manually added physical activities when they knew the ring would not record correctly. Many women were not interested or engaged in using the physical activity features of the system as they felt the advice and recordings were not in agreement with their own goals (expressed as either too much asked of them or too little).

Many women in the high-use group had past experiences with self-monitoring equipment. This led some to compare devices, and some used this previous familiarity in self-monitoring to get the best out of the service through paired use with other devices. Some women expressed that they struggled to always wear this ring because the size and style appears to be best suited to men or it was too big and sometimes caught on items or their children during handling.

### Mediation Activities of the eHealth System

The eHealth system presented new health resources in combination with women’s activities. Nurses and women developed new patterns of interacting due to access to data that was collected in between clinic visits. With the help of their PHNs, perinatal clients developed a personal understanding of the possible links between their health behaviors and their health states in a way that was not possible before having access to on-demand data.

#### Gentle and Guiding Nature of eHealth Advice: High-Use Group

Women in the high-use group expressed that the app was not mean or rude in its recommendations and tips and that having access to health behavior data gave them and their PHNs insight into the connection between health behavior and stress management. ŌURA reminds its users about balancing activity and rest and explains how this could improve overall stress outcomes through use of its proprietary recovery score reports. Women in the high-use group noted that whether the recordings are accurate or not, interfacing with the system provided an opportunity for interpreting overall progress on matters such as stress, sleep, and physical activity with nurses. One woman articulated this when asked whether the eHealth system would fit into maternity clinic care:

Well, why not…Like on very many things connected also to health, body functioning, and other, I think it would fit very well.

Participant 18

Women in the low-use group did not express a perception of the eHealth system as being a guide or a gentle service for them; they did, however, discuss that looking at the data with the nurses during the clinic visits was an enjoyable and nice experience for them because the nurses used the system to discuss things that were not acute. Further, women mentioned that the nurses did not use a judgmental tone when discussing the personal data in the clinic.

#### Flexible and Responsive Tips and Information

The app was responsive in some respects to women’s daily patterns and available on demand. This provided women with an opportunity to view data according to their interest and energy levels at any given time. The information was categorized into sleep, stress, and physical activity separately, and women could look up information about each topic. Women suggested that this structure allowed for them to participate in health promotion activities when and how they wanted. Some women stated that the high volume of options made it a bit hard to get used to using the system initially, and after getting comfortable with their own use threshold, they were satisfied. Some women thought it was interesting to view data as questions in their life arose; in 1 case, a woman and her partner were wondering about sleep latency and were able to answer their questions with the system right away.

#### eHealth Provided New Opportunities for Participation

Participants mentioned that they had new and interesting experiences during their clinic visits with the nurse because of using the system. Women gained new accountability in their care team because they could contribute to the care planning with self-monitoring data. These data provided more information about daily life contexts and could be used in the interpretations of health states of the women. Further, the women and nurses had experiences of looking at the same data and working out interpretations together. Viewing data together also gave some women an opportunity to share their emotional struggles that related to the patterns that they might otherwise not have. Some women took the lead; in 1 case, the woman showed the nurse the data on her phone because the nurse could not log on to her computer system.

## Discussion

### Principal Findings

Our study revealed that pregnant users participated in the cocreation of the use of an eHealth self-monitoring system through the presence of adaptive (the women) and mediating (the eHealth system) activities. What is novel in our study is the use of a sociomaterial perspective and the exploration of the influences of different behavioral engagement levels (physical wear time of the device) in the development of the meaningful use of an eHealth system. Identifying and defining the activities of both perinatal users (high- and low-use groups) and the eHealth system itself illuminated interactions and processes that inform designs based on our user groups’ skills, habits, and values.

A recent review noted that of 12 different emerging perinatal eHealth modalities, self-monitoring was the third-most prevalent in use in the developed world [24]. A common reason for providing access to eHealth self-monitoring systems was to promote the movement of perinatal service away from the clinic into the home environments of clients [25-28]. Researchers report that by moving care from the clinic spaces back into the homes of perinatal clients, care inequities and power imbalances between providers and clients could be ameliorated [26,29]. Self-monitoring is trending with the expressed goal of improving patient engagement and perinatal health outcomes; however, there is limited examination of the meaningful use of eHealth systems [24,30]. Not all high use was related to meaningful use for the pregnant users in our study. Some women used the system a lot but did not see the need to develop goals related to their lifestyle, while others did not consistently record their activities and sleep but found discussions with PHNs about incidences of poor sleep useful for managing their well-being during pregnancy.

What is lacking in many studies on evaluating perinatal self-monitoring systems is a clear definition and examination of patient engagement and associated health outcomes [24]. A descriptive comparative analysis conducted as part of our larger feasibility study revealed the limitations of only examining the physical use of self-monitoring technology [18]. The process characteristics will be missed if we do not look beyond physical use as an indicator of engagement [18]. Here, women cocreated their use patterns aided by the mediation of the eHealth system. Use patterns were cocreated based on personal preferences and attitudes about technology and the adaptive qualities of the eHealth system to pregnant users’ circumstances. For example, in some cases, the system aided in their receipt of appropriate resources in the form of tailored feedback (ie, notes about relaxing a little during the day because of poor sleep).

Participants experienced a process of adapting in the use of the system, and they were able to obtain benefits from the self-monitoring program with low or high amounts of engagement, depending on their preferences or life circumstances. Experts in the field of eHealth design have pointed to the impact personal habits, routines, and life skills can have on one’s choice and patterns of the use of eHealth technologies [31]. What is yet to be deeply explored is how these variations can support personalized effective “dosing” of engagement in eHealth system use, and many studies report under the notion that “the more the use, the better” [32]. A study was conducted to evaluate the feasibility of building unique profiles within an eHealth system created by researchers using The Incredible Intervention Machine (TIIM) to understand the impact of personalization based on the client’s intended use and engagement in the intervention [33]. Directions toward conceptualizing our programs through the threshold for intended use have been supported by other researchers; in a systematic review of eHealth evaluations, authors highlighted that historically, pharmaceutical influence supports the concept of intended use and this spills over into general health program development fields [32]. The assumption that eHealth users should reach a standard level of intended use for effectiveness to be achieved was then perpetuated in the field. In our study, it is apparent that users found individual effective thresholds of use. The unique experiences of receiving support, encouragement, and coaching toward health lifestyle habits that are tailored specifically for each perinatal patient can have lasting positive impacts on health promotion and self-care regardless of a specific eHealth program dose.

Women in the high-use group suggested using the service beyond pregnancy during the postpartum period, and all women in our study said they had an affinity with using technology in their lives as information support. This indicates that the adapting process described in the findings of our study is unique to pregnant women who find technology beneficial to some degree and choose their own intended usage in order to match the technology to their own goals. Experts in eHealth user engagement have suggested designing programs, keeping in mind the dose-response relationship rather than the adherence-response relationship, that is, “the more use, the better” [32]. Our study illustrates that in populations and contexts where pregnant persons are open to interacting with eHealth at some level, it is possible to identify user-specific intended usage.

### Limitations

This study was conducted to understand the feasibility and useability of an eHealth self-monitoring system examined through a pilot use of the system. The sample was small, and the eHealth modalities of ŌURA ring 3.0 have changed to incorporate new functionalities that the women in our study would have liked to see at the time of our pilot use in spring/summer 2020. The findings of this study do not inform the effectiveness or efficacy of such an eHealth system but do inform on strategies and the research directions to take in the future development of personalized eHealth self-monitoring systems. We were not able to note any clear link between the demographics of our participants and their level of use. The ability to wear the smart device at work might have played a role in the low-use group; however, our study took place when there was a governmental stay-at-home order and some of the participants were taking vacation time during the period of the study. We recommend that future research be conducted with a larger group and a specific focus on links between demographic variables and use patterns, habits, and values. Further, our study was limited in examining real-time reactions of our participants to the app’s automatic cues and tips given in real time and on demand. For our chosen system, this was not possible, and we recommend that to learn about real-time adapting to behavior change in the future, researchers integrate a way to monitor this type of real-time response to automatic behavioral prompts.

### Conclusion

This feasibility study highlights the value of examining the processes of adapting in the pilot use of a perinatal eHealth self-monitoring system. Women in our study had varying levels of use and cocreated their eHealth system use along with the technology mediation. Mediating activities conducted by technology play an important role in the restructuring of perinatal care programs and have potential for improving personalization and accessibility of antenatal resources. The exploration of the meaningful use of eHealth systems is recommended as pregnant users’ ability to obtain appropriate health resources depending not only on having systems accessible to them but also on their own use patterns that are based on personal preferences, values, and habits.

## Abbreviations

PHN: public health nurse

## Data Availability

The participants of this study did not provide written informed consent for their data to be shared publicly, so due to the sensitive nature of the research, supporting data are not available. Qualitative data from this study are not available in raw form; however, the codebook and coding details can be shared upon request.
